# Event and Comment

**Published:** 1909-06-15

**Authors:** 


### DENTAL REGISTER.

**Vol. LXIII. June 15, 1909. No. 6.**

### EVENT AND COMMENT.

SILICIOUS CEMENTS. We print in this issue three papers which fairly set forth this subject, which just now seems to be attracting the attention of the profession more seductively than any other. The results of careful manipulation produces results that certainly seem to warrant giving the material a full investigation. There is no question but the fillings have a live tooth appearance never before secured with other cements, and they are so hard as to resist mechanical abrasion. In fact this very hardness seems to be one quality which is likely to cause failures at the margins unless great care is taken to secure proper cavity preparation which will obviate thin projections of the material which are liable to chip away and form a good place for recurrent caries. If we can get away from the present ideas of cavity preparation for metallic fillings, which require thick margins, and adopt methods which will preserve the thin margins, we shall probably realize better results. There has as yet been insufficient time to determine what effect the oral secretions shall have in dissolving this material as it does the oxyphosphates. This tendency at present is not marked. Careless manipulation has already brought out the fact that it must be handled with the strictest obedience to cleanliness of manipulation and with non-corrosive instruments, or unsightly color problems follow. No dust or dirt of any kind, or acids or metals can be mixed into the mass without becoming conspicuous after a few months wear. The material sets so hard and so promptly that it must be handled rapidly and yet it must be given considerable time to set. Many dentists have accepted it as a cheap and quick working material, but we find that the time required for its manipulation is not materially shorter than for a gold or alloy filling, consequently it is not a cheap substitute. It is true it can be manipulated into the cavity with more ease than gold, but it is much more difficult to fill a tooth with it than amalgam or cement. The classes of cavities to which it is most applicable are those usually on exposed surfaces, and which would need to be filled with porcelain; such as labial surfaces of exposed teeth, and proximal surfaces of the same teeth, it is especially indicated for small proximal cavities which may be reached with slight separation and little cutting through to the labial surfaces. It also can be used advantageously for cervical cavities encircling the roots considerably exposed by gum recession. The material should be mixed according to directions or it becomes granular and brittle, and as it does not adhere to dentine considerable retention form should be provided in the cavity. The longer it is kept free from moisture while crystalizing the better it sets. The profession, it seems to us, is justified in expecting satisfactory results from this material but we are pleased to notice that every one seems to be using it with a conservatism that is prudent.

